# Positive Selection in the Light Zone of Germinal Centers

**DOI:** 10.3389/fimmu.2021.661678

**Published:** 2021-03-31

**Authors:** Rinako Nakagawa, Dinis Pedro Calado

**Affiliations:** ^1^ Immunity and Cancer Laboratory, The Francis Crick Institute, London, United Kingdom; ^2^ Peter Gorer Department of Immunobiology, School of Immunology & Microbial Sciences, King’s College London, London, United Kingdom

**Keywords:** positive selection, cMyc, affinity maturation, permissive selection, clonal diversity

## Abstract

Germinal centers (GCs) are essential sites for the production of high-affinity antibody secreting plasma cells (PCs) and memory-B cells (MBCs), which form the framework of vaccination. Affinity maturation and permissive selection in GCs are key for the production of PCs and MBCs, respectively. For these purposes, GCs positively select “fit” cells in the light zone of the GC and instructs them for one of three known B cell fates: PCs, MBCs and persistent GC-B cells as dark zone entrants. In this review, we provide an overview of the positive selection process and discuss its mechanisms and how B cell fates are instructed.

## Introduction

Germinal centers (GCs) are sites where antibody affinity for the antigen (Ag) is improved and Ag-activated B cells differentiate, hence they are important for host defense and clearance of exogenous pathogens. This specialized microstructure transiently forms within the B cell follicles of secondary lymphoid organs during the course of T cell-dependent immune responses. The process of increasing antibody affinity is known as affinity maturation (1, 2) and results from somatic hypermutation (SHM) of immunoglobulin (Ig) genes in GC-B cells and clonal selection (3). GCs include two distinct regions, light zone (LZ) and dark zone (DZ) (4). SHM is mediated by activation-induced cytidine deaminase (AID) (5) and occurs in the DZ where GC-B cells extensively proliferate. In the LZ, GC-B cells are selected in an Ag and T cell-dependent manner. LZ-B cells retrieve Ag on follicular dendritic cells (FDCs) that can uniquely retain and display Ag in the form of immune complex (ICs) (6). B cell receptor (BCR) binding of Ag by LZ-B cells results in internalization of BCR-Ag and subsequent presentation of Ag in the form of Ag-specific-peptide-major histocompatibility II (pMHCII), which enables them to receive help from T follicular helper cells (TFHs). These positively selected LZ-B cells induce cMyc, a critical regulator for GC maintenance and proliferation, and cMyc positivity transiently marks “licensed” GC-B cells (7, 8). cMyc^+^ GC-B-cells in the LZ re-start the cell cycle and travel to the DZ for further cell division (7–9). GC-B cells undergo iterative rounds of mutation and selection through a migration cycle between LZ and DZ. Eventually, GC reactions produce high-affinity antibody secreting plasma cells (PCs) and memory-B cells (MBCs). In this review, we summarize and discuss studies illustrating how positive selection of GC-B cells are triggered, what molecular and cellular events that GC-B cells undergo during the process of positive selection, and how B cell fate decisions are coordinated during positive selection.

## Mechanisms By Which GCs Positively Select LZ-B Cells

### Current Models for Affinity-Dependent Positive Selection

In response to signals from BCR engagement and TFHs, a fraction of LZ-B cells are positively selected and results in evasion of apoptosis partially in a microRNA-155-dependent manner (10, 11). cMyc is induced upon positive selection and its expression effectively defines positively selected GC-B cells (7, 8). In the currently favored model, positive selection occurs in an affinity-dependent manner (12, 13). LZ-B cells capture FDC-bound Ags through their BCRs, process and present them in the form of pMHCII and signals downstream of BCR-Ag engagement allow survival. Higher-affinity GC-B cells more effectively receive helper signals from TFHs because they acquire more Ag, present pMHCII at higher levels and thereby induce greater TFH activation, this is in line with studies from early B cell responses *in vivo* (14) and *in vitro* (15). For promoting efficient positive selection, recycling GC-B cells reset their BCRs and MHCII before reentering the LZ (16, 17). Contact duration between cognate T cells and GC-B cells is shorter than that between T cell and Ag-activated B cells before GC formation (12, 18, 19). Moreover, only a limited proportion of T cells in GCs appear to actively interact with GC-B cells that are significantly more numerous than TFHs within the time window of confocal microscopic analysis (12, 18). These observations suggest that interactions between GC-B cells and TFHs are strictly controlled and therefore GC-B cells may compete for cognate T cell help. Together with a mathematical simulation model (20), these findings support that T cells are a limiting factor and positive selection can occur in a T cell-driven selection mechanism (12, 21). This selection mechanism is further supported by studies using a DEC-205-antibody-based Ag delivery approach (22). DEC-205 is an endocytic receptor that is primarily expressed in dendritic cells but also in B cells and directs captured Ag to Ag-processing compartments (23). Administration of Ag coupled to anti-DEC205 antibody allows delivering the Ag to endosomal compartments independently of BCR *via* a DEC205 receptor in GC-B cells (24) and results in greatly enhanced presentation of Ag peptide regardless of the nature of BCR (25). DEC-205-antibody-mediated Ag delivery prolongs interactions between GC-B cells and TFHs (26) and enables GC-B cells to gain more help from TFHs (9, 13, 24). Consequently, transcript levels of cMyc are considerably increased in the DEC205 agent-treated GC-B cells in a dose dependent manner (7, 27). The series of experiments demonstrate that providing strong T cell help to the total GC-B cell population during GC responses greatly potentiates positive selection process and resultant proliferation (24, 27–29). These findings underscore essential roles of T cell help in positive selection. However, a recent report has shown that by interrogating NP-specific GC-B cell responses in MHCII haploinsufficient mice in which both MHCII and pMHCII are halved compared to WT mice, the density of pMHCII is not as critical for selection in established GCs as in naive B cells (30). This finding suggests that other factors also play a role in an affinity-dependent positive selection, such as BCR signaling.

Strong BCR signaling through soluble Ag binding eliminates Ag-specific GC-B cells by inducing apoptosis predominantly in LZ-B cells within hours of engagement (31–33), thus enhanced BCR signaling is deleterious for GC-B cells. In agreement with this, canonical BCR signaling pathways are attenuated in GC-B cells compared to those in naïve B-cells (34–37) due to negative feedback by the activation of negative regulators for BCR signaling in a phosphate and tensin homolog (PTEN)-dependent manner (38). Nonetheless, GC-B cells can transmit PI3K-mediated signaling through BCR in a Syk-dependent manner to restrict the activity of forkhead Box O1 (FoxO1) (39), a critical transcription factor for the DZ transcriptional program (25, 40, 41). This occurs through synaptic interactions between GC-B cells and FDCs in which GC-B cells can respond to membrane-bound Ags more efficiently than naïve B cells in an affinity-dependent manner (36, 37, 42). Using an adoptive transfer system, the propagation of donor derived GC-B cells is investigated upon restimulation with sub-saturating T cell help provided by DEC205 agent in the presence or absence of simultaneous Ag injection. Restimulation with Ag in the presence of T cell help enhances GC responses compared to T cell help only (29), suggesting that BCR signaling could potentiate positive selection synergistically with T cell help. Transient BCR signals prime B cells and alter their nature for forthcoming contact with T cells prior to GC formation (43, 44). Similar alterations might occur in GC-B cells upon reception of BCR signals.

### Potential Alternative Mechanisms of Permissive Positive Selection and the Beyond.

The permissive environment of GCs confers clonal breadth in MBCs that is effective for viral clearance (45, 46). Recent findings underpin that GCs permit retaining cells with varied affinities for the antigen (47–49). Permissive positive selection cannot be well explained only by affinity-dependent models and there could be an alternative mechanism that is not well understood. We recently identified cMyc^+^ LZ-B cell subpopulations that arise at different times following the reception of positive selection signals. Analysis of the cMyc^+^ LZ-B cell subpopulations revealed that a significantly large fraction of low-affinity cells is initially positively selected, but are mostly outcompeted by more proliferative higher-affinity cells before DZ migration (50). This mechanism partially explains how low-affinity GC-B cells can be retained in GCs because positively selected GC-B cells can avoid apoptosis in GCs (10, 50). In our observations, rather than selecting only higher-affinity cells from the beginning of positive selection, GCs permit selection of low-affinity cells at the beginning of each round of positive selection, thus selection is relatively independent of BCR affinity. This mode of positive selection continues for a period of time during the height of GC reactions (50). However, the differential strength of signals between low-affinity and higher-affinity cells eventually leads to differential proliferation rate of positively selected cells. As a result, higher-affinity cells proliferate better and are enriched in cMyc^+^ LZ-B cells compared to cMyc^-^ LZ-B cells (7, 50). Following Ag-mediated activation, over time Ag may become more limited with GC-B cells having more competition for antigen-induced survival signals. If GC-B cells are fit enough despite carrying low-affinity BCRs, they could compete effectively for Ag and acquire Ag successfully.

We noted that CD40 expression is elevated in the cMyc^+^ LZ-B cell subpopulation emerging soon after positive selection compared to the cMyc^-^ LZ compartment that contains GC-B cells before positive selection; although this subpopulation largely comprises low-affinity cells at a similar level to that of cMyc^-^ LZ-B cells (50). Increased CD40 expression might allow these GC-B cells to gain more T cell help by these two mechanisms; i) inducible T cell co-stimulator ligand (ICOSL), a ligand for ICOS that is one of the co-stimulatory receptors expressed on TFHs, is induced on GC-B cells through CD40 engagement with CD40L on TFHs and potentiates GC-TFH interactions (51, 52); ii) these cells may have an increased chance of interacting with IL-4 expressing TFHs that express CD40L at a higher level than IL-21/IL-4 expressing TFHs or IL-21 expressing TFHs (53). Alternatively, reduction of the engagement of Herpesvirus entry mediator (HVEM) on GC-B cells with a ligand, B- and T-lymphocyte attenuator (BTLA) on TFHs leads to increased expression of CD40L in TFHs (54). Thus, reduced expression of negative regulators such as HVEM on GC-B cells can also lead to an increased amount of T cell help. The underlying mechanisms for upregulation of CD40 in these GC-B cells are unknown, but they perhaps receive additional signals prior to, or at the initiation of, receiving cMyc-inducing signals to upregulate the molecule. CD40 expression on B cells is shown to be enhanced *in vitro* by B cell-activating factor (BAFF) (55) which can be secreted by FDCs (56, 57). Under the condition that only a limited amount of Ag is available, GC-B cells that successfully acquire Ag could receive survival signals from FDCs *via* contact-based interaction and/or *via* trophic factors independently of BCR affinity. This could provide an advantage for GC-B cells to undergo positive selection [functions of FDCs during GC responses are extensively discussed elsewhere (58–60)]. In such case, modulation of FDCs’ functions by other factors, such as Toll-like-receptor (TLR) 4 ligands (61), may play a role in permissive selection.

**Figure 1:**
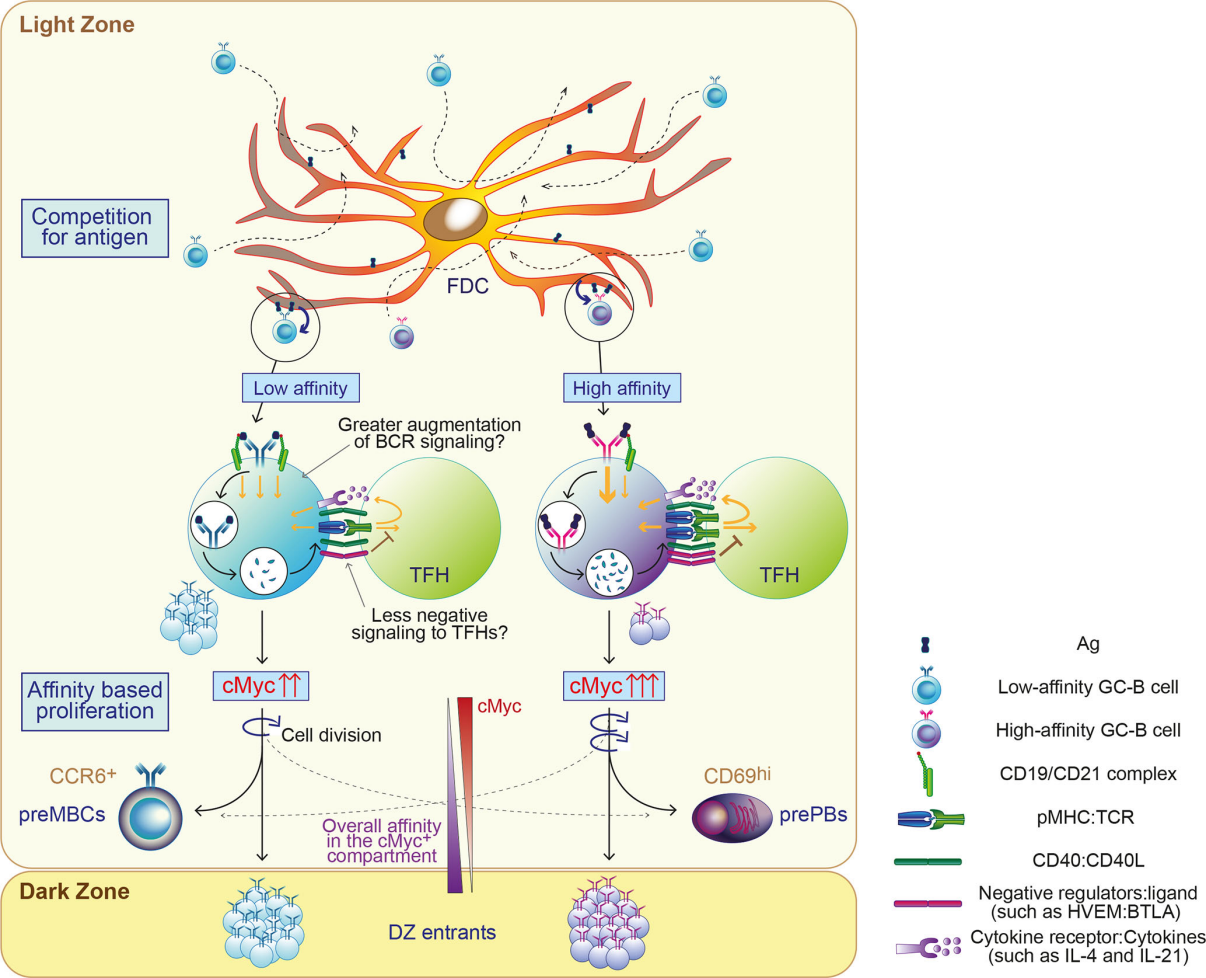
Proposed model of permissive positive selection in GCs. GC-B cells compete for antigen when only limited amount of antigen (Ag) is available. Fit cells retrieve Ag deposited on follicular dendritic cells (FDCs) relatively independently of BCR affinity and receive survival signals from FDCs through contact-based interaction and/or trophic factors. Both high and low-affinity cells process Ag and present differing levels of Ag in the form of peptide-MHCII complex (pMHC) in proportion to its affinity. Low-affinity cells may augment the level of signaling above a threshold with potentially undefined mechanisms, such as favorably augmented signals and/or less negative feedback. GC-B cells received sufficient signals for positive selection proliferate mainly based on their BCR affinity. Their fates are also instructed, generally depending on their BCR affinity. cMyc^+^ GC-B cells divide in the LZ and cMyc expression in GC-B cells is reduced accordingly. The width of the arrows in GC-B cells and TFH depicts the signal strength.

The concept that higher-affinity cells are favorably selected from the beginning of the positive selection process is supported by *in vitro* microscopic observations that the higher-affinity cells can form more stable contacts with the membrane, better resist the pulling force required to capture Ag without causing cell rupture, transmit stronger BCR signals and acquire a larger amount of Ag to present to T cells compared to low-affinity cells (36, 37, 42, 62). However, the proportion of higher-affinity cells in the cMyc^+^ subpopulation that appears soon after positive selection is similar to that of cMyc^-^ LZ compartment before positive selection (50). This suggests that low-affinity cells can be selected at a comparable level as higher-affinity cells at the beginning of positive selection. The discrepancy could be caused by the method of Ag presentation by FDCs, since multimerized Ag on FDCs can impact the GC selection process (59, 63) and also by the availability of complement proteins (such as C3d) that are required for bridging the Ag bound to BCR and the BCR coreceptor complex. The BCR co receptor complex consisting of CD19, CD21 (a.k.a. complement receptor type 2, CR2) and CD81 can augment BCR signaling and Ag processing, to lower the Ag threshold and quicken Ag presentation (64–66). Potentially, the differential expression of BCR coreceptor complexes and/or negative regulators such as HVEM on low-affinity cells may allow them to be more competitive with higher-affinity cells by enhancing BCR signaling and T cell help (**Figure 1**). More investigation is required to elucidate mechanisms allowing low-affinity B cells to be positively selected.

Recent reports have shown that DEL-OVA (duck egg lysozyme-ovalbumin) *ex vivo* pulsed DEL-specific HyHEL-10 B cells can join existing GCs elicited by OVA (i.e., DEL has not been deposited on FDCs) and contribute to GC responses at a comparable level to DEL-OVA-immunized recipients (44, 67). In these experiments, the HyHEL-10 B cells that were exposed to DEL-OVA *ex vivo* for 5 min were transferred into recipient mice that had been immunized with OVA for 3 or more days. This may be interpreted as single Ag acquisition by B cells is sufficient for them to participate in GC responses without any further Ag acquisition; alternatively, the HyHEL-10 B cells pulsed for 5 min might somehow deliver un-internalized Ag to FDCs and the deposited Ag may have been used for robust GC responses. It has been controversial whether retention of immune complex (ICs) on FDCs is essential for GC formation and positive selection (68–71), while reports have shown that ICs deposited on FDCs during GC responses contribute to optimum affinity maturation (72–74). Nonetheless, the results using HyHEL10 B cell adoptive transfer system suggest two possibilities; i) GC-B cells can survive and proliferate with very little Ag if B cells have taken up Ag adequately during initial activation; and ii) B cells activated by a dissimilar Ag to the original GC initiating Ag may take over the GC if the newcomer B cells receive cognate T cell help (in this case, transferred DEL-specific B cells can present OVA-peptide and thus can receive T cell help from OVA-specific T cells in OVA-elicited GCs). Inter-GC trafficking of B cells into neighboring GCs is suggested by long-term observations of single GCs using intravital microscopy technique (47). Since GCs are dynamic open structures and TFHs can emigrate into neighboring GCs (75, 76), it is still possible that a GC-B clone recognizing an Ag re-seeds in neighboring GCs whose reactions are elicited by another Ag (or cryptic epitopes) if cognate T cells also migrate. A recent report has shown that GCs elicited by complex Ags somehow permit B cells that do not detect the original Ag (48). Retention of varied clones in GCs may be attained by a combination of inter-GC trafficking and intra-clonal permissive selection.

## Molecular Events During Positive Selection and B Cell Fate Instruction

The cMyc^+^ GC-B cell compartment is suggested to be heterogeneous due to differential signal activation (25, 41) and as a result gives rise to heterogeneous populations containing future PBs/PCs, DZ-entrants and future MBCs (24, 50, 77).

### Fate 1: Plasmablasts/Plasma Cells (PBs/PCs)

Increased TFH help drives GC-B cells to differentiate into PBs/PCs (24, 29, 77) with enhanced NF-κB signaling *via* CD40-CD40L ligation (39, 78, 79). Consistent with these findings, PB/PC precursors in GCs defined as cMyc^+^ CD69^hi^ Bcl6^lo^ LZ-B cells express relatively high levels of IRF4 (50, 77) which is a critical transcription factor for PC differentiation and induced by NF-κB signaling (80). These distinct precursors consist of high-affinity GC-B cells in agreement with previous findings (81, 82) and become detectable soon after positive selection (50, 77). Stable contact between TFHs and GC-B cells as a consequence of greater pMHC presentation in GC-B cells induces Ca^2+^-dependent expression of IL-21 and IL-4 in TFHs (26). Thus, stronger T cell help inducing signals promote PB/PC differentiation from GC-B cells by producing cytokines that support PC differentiation (53). However, some GC-B cells persistently remain as GC-B cells (i.e., become DZ-entrants) instead of differentiating into PBs/PCs upon receiving exogenous strong T cell help by DEC205 agent (24, 28), suggesting that there is still a missing component for the PB/PC fate instruction other than TFH help. Recent reports have shown that signaling induced by Ag-BCR engagement contributes to PB/PC differentiation from GC-B cells (29, 44, 81, 83, 84). Strong BCR signaling in conjunction with CD40 signaling can upregulate IRF4 by degrading the E3 ubiquitin ligases Cbl that ubiquitinates IRF4 for degradation in GC-B cells (84). PB/PC output is largely influenced by Ag valency that reduces Ag affinity threshold in extrafollicular PC responses (85, 86), thus multivalent Ag presentation on FDCs may also play a role in PB/PC differentiation from GC-B cells.

### Fate 2: Memory-B Cells (MBCs)

In contrast to PBs/PCs, MBC precursors predominantly contain lower-affinity cells and appear to require only minimal amount of help from TFHs (87, 88). Together with the observations that MBC precursors are relatively quiescent (87–89), it is broadly assumed that MBCs arise from “non-positively-selected” LZ-B-cells. However, this concept cannot explain these three points; i) how the specificity of the BCR can be checked before positive selection, ii) how cell survival can be assured without positive selection, and iii) how high-affinity clones can be selected for MBC differentiation from a pool of GC-B cells with varied affinity. To understand the origin of MBCs, extensive single cell transcriptomic analysis identified a preMBC subset in mouse spleens (90) and human tonsils (91). These preMBC subsets are characterized by the expression of *Ccr6* (90, 91) as expected from a previous report (88). Unexpectedly the mouse preMBC subset has relatively higher *Mki67* expression than MBC subsets (90) and the human preMBC subset exhibits a similar level of *Myc* expression to subsets containing positively selected cells (91), which is counter-intuitive considering the relatively quiescent nature of MBC precursors as previously reported (87–89). These findings suggest that CCR6^+^ preMBCs could receive sufficient signals from TFHs to induce cMyc and divide before fully differentiating into MBCs. In agreement with these findings, we identified MBC precursors within cMyc^+^ positively selected GC-B cells that are less proliferative than other cMyc^+^ LZ-B cells but still dividing (50). Stronger TFH help induces differentiation of CD80^hi^ MBCs whose Ig genes accumulate mutations compared to those of CD80^-^ MBCs (92, 93), and resultantly CD80^hi^ MBCs comprise relatively high-affinity clones (93). A very small fraction of high-affinity cells within the cMyc^+^ MBC precursor subpopulation (defined as cMyc^+^ CD23^hi^ CCR6^+^ LZ-B cells) (50) might differentiate into CD80^hi^ MBCs following reception of robust T cell help. For the MBC fate instruction, interplay between transcription factors exerts functions that regulate MBC differentiation from cMyc^+^ GC-B cells. Myc-interacting zinc finger protein-1 (MIZ-1) is expressed in most cMyc^+^ LZ-B cells and the transcriptional activator Miz-1 switches to a transcriptional repressor upon cMyc binding (94, 95). The cMyc/Miz-1 complex represses Miz-1 target genes and results in restricting positively selected GC-B cells from forming MBCs to favor GC-B cell fate as DZ-entrants (94). A transcription factor, hematopoietically-expressed homeobox protein (Hhex) that is expressed in MBC precursors and promotes MBC differentiation (50, 90) can interact with cMyc to decrease its activity, including cell proliferation and metabolism in tumors (96). Reception of differential signals for positive selection induces these transcription factors at varied levels in positively selected GC-B cells (97), which most likely play a part in MBC differentiation. However, the overall nature of the signaling network for transition from GC-B cell to MBCs remains unknown.

### Fate 3: DZ Entrants

The remaining positively selected LZ-B cells other than cells instructed to PB/PC and MBC fates transit to the DZ for further proliferation as DZ-entrants. BCR signals downregulate FoxO1 and cyclin D3, which are essential for maintenance and proliferation of DZ-B cells, respectively (25, 39–41, 98, 99). Strongly induced cMyc expression in positively selected LZ-B cells in turn activates activating enhancer binding protein 4 (AP-4) that contribute to the induction of cyclin D3 (100). Hence, positively selected cells are likely to turn on the DZ-proliferation program when BCR-induced signals are weakened in the LZ, which is concordant with previous observations about the co-expression of FoxO1 and/or cyclin D3 together with cMyc in positively selected LZ-B cells (41, 98, 100).

## Concluding Remarks

Clonal breadth achieved by permissive selection is particularly useful for protection from viruses that constantly mutate. Understanding the underlying mechanisms of permissive selection followed by B cell differentiation will guide vaccine design and improve their efficacy in the future.

## Author Contributions

RN and DC wrote the manuscript. All authors contributed to the article and approved the submitted version.

## Funding

This work was supported by the Francis Crick Institute, which receives its core funding from Cancer Research UK (FC001057), the UK Medical Research Council (FC001057), and Wellcome Trust (FC001057) to DC and by the UK Medical Research Council (grant reference MR/J008060) to DC.

## Conflict of Interest

The authors declare that the research was conducted in the absence of any commercial or financial relationships that could be construed as a potential conflict of interest.
